# A Case of *Plasmodium malariae* in Bangladesh: A Representation of the Suboptimal Performance of Rapid Diagnostic Approaches in Malaria Elimination Settings

**DOI:** 10.3390/pathogens11101072

**Published:** 2022-09-21

**Authors:** Fatema Tuj Johora, Mohammad Golam Kibria, Hans-Peter Fuehrer, Mohammad Shafiul Alam

**Affiliations:** 1 International Centre for Diarrhoeal Disease Research, Bangladesh (icddr,b), Dhaka 1212, Bangladesh; 2 Institute for Biomedical Sciences, Georgia State University, Atlanta, GA 30303, USA; 3 Department of Medical Biochemistry, Faculty of Life Sciences, Kumamoto University, Kumamoto 860-8556, Japan; 4 Institute of Parasitology, Department of Pathobiology, University of Veterinary Medicine Vienna, Veterinaerplatz 1, 1210 Vienna, Austria

**Keywords:** malaria, *Plasmodium malariae*, rapid diagnostic test, malaria elimination, Bangladesh

## Abstract

*Plasmodium malariae* is a neglected human malaria parasite with low parasitemia that often results in the misdiagnosis and underestimation of the actual disease burden of this pathogen. Microscopy is the best diagnostic tool, despite the fact that rapid diagnostic tests (RDTs) are the best surveillance tool for malaria diagnosis in many rural areas for their ease of use in elimination settings. For parasite antigen detection other than *P. falciparum*, RDTs depend on essential glycolytic *Plasmodium* proteins, i.e., *Plasmodium* lactate dehydrogenase (pLDH) and *Plasmodium* aldolase (pAldo) antigens. There is a lack of species-specific test kits for *P. malariae*, and overall, its rapid antigenic test accuracy is questionable. False negative results can accelerate the burden of asymptomatic malaria infection and transmission. Here, we report a case of a malaria patient in Bangladesh infected with *P. malariae* who tested negative on pLDH and pAldo based RDTs. This case provides useful information for health providers to be aware of possible RDT failure and also for the future development of analytically sensitive test kits for *P. malariae.*

## 1. Introduction

*Plasmodium malariae* is one of the six parasites causing human malaria. Because of its low parasitemia and lower pathogenicity, it is often neglected. This protozoan parasite has been observed in all malaria-prone regions of the world [1]. In Bangladesh, *P. falciparum* is the dominant and most relevant species, but *P. malariae* is present as well [2,3,4,5]. A cross-sectional study based on molecular diagnosis showed a prevalence of 2.7% of *P. malariae* in the Chittagong Hill Tracts, but this parasite was documented in 9.5% of all malaria-positive asymptomatic participants [3]. Infections can be prolonged and subclinical [6]. Rarely, infection can result in renal dysfunction [7]. Moreover, the transmission and pathogenesis of other malaria-causing species can be influenced by this parasite [8]. Species-specific therapeutic administration is very crucial in the management of malaria cases and for eradication programs. There are also reports of therapeutic failure in chronic multi-species infections [9,10]. For the case detection of malaria in remote areas, community health systems depend mainly on rapid diagnostic test (RDT) kits because of their accessibility and readiness in case detection [11]. Malaria RDTs are based on various *Plasmodium* antigens, such as *P. falciparum* histidine-rich protein-2 (PfHRP2) for the diagnosis of *P. falciparum* only, and *Plasmodium* lactate dehydrogenase (pLDH) for the diagnosis of all *Plasmodium* species (pan-specific) or specifically *P. falciparum* (PfLDH) and/or *P. vivax* (PvLDH). Moreover pan-specific *Plasmodium* aldolase (pAldo) RDTs are available. However, none of the antigen detection kits is specific for *P. malariae*, *P. ovale* spp., or *P. knowlesi* [12]. Today, microscopy is still recommended as the gold standard for these neglected human malaria parasites, but it might result in false negative results with low parasitemia or in mixed infections. In areas where *P. knowlesi* is endemic, it remains problematic to distinguish *P. malariae* from *P. knowlesi* due to their similar morphology [13]. Hence, detection is considered a challenge in many endemic settings.

In this case, we report an infection with *P. malariae* that tested negative on RDT (based on PfHRP2/pLDH) in Bangladesh despite the presence of a relatively high parasitemia. 

## 2. Case Report

**Figure 1:**
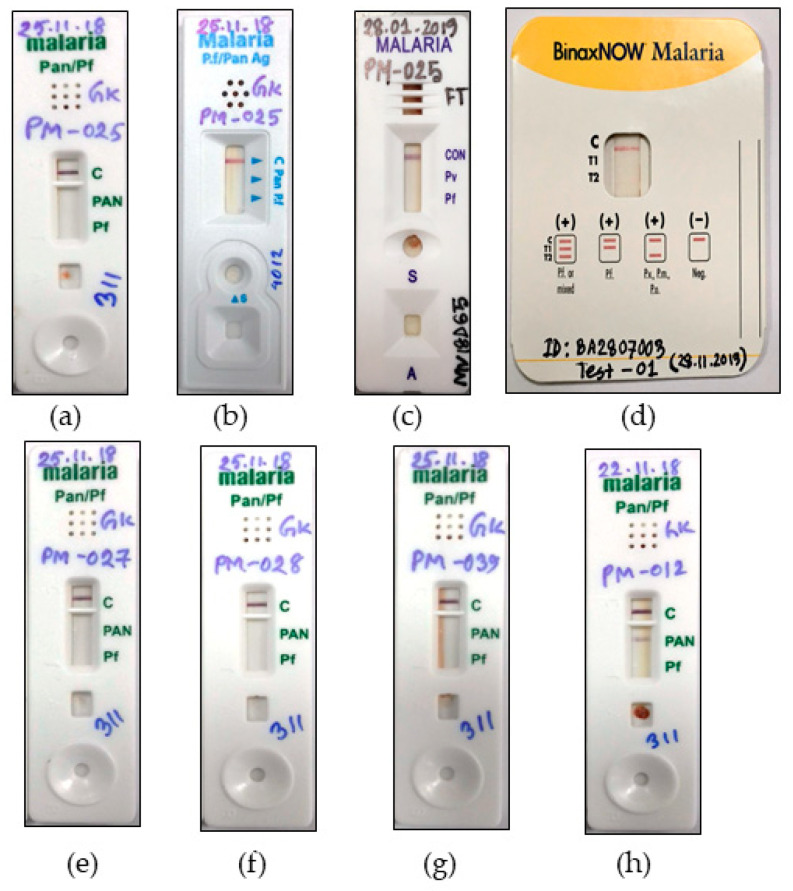
RDT kits used for the diagnosis of *P. malariae*: (**a**) case sample (PM-025): 5930P/µL (Parascreen^TM^ Rapid Malaria Pf/Pan); (**b**) STANDARD Q Malaria P.f/Pan Ag; (**c**) CareStart™ Malaria HRP2/pLDH (Pf/Pv) Combo; (**d**) BinaxNOW^®^ Malaria Test.; (**e**–**g**) diluted case sample (PM-027): 4500P/µL; (PM-028): 3000P/µL, and (PM-039): 2000P/µL (Parascreen^TM^ Rapid Malaria Pf/Pan); (**h**) positive control (PM-012): 5040P/µL (Parascreen^TM^ Rapid Malaria Pf/Pan).

In July 2018, a blood sample was obtained from a 15-year-old male who presented with high fever, chills, nausea, and headache at the Alikadam Upazila Health Complex (AUHC) in Bandarban, Chittagong Hill Tracts, Bangladesh (92.3120° E longitude and 21.6523° N latitude). The patient resided near the AUHC and reported no travel activity in the past three months outside his territory. At the AUHC, the sample tested negative on RDT using a CareStart™ Malaria HRP2/pLDH (Pf/Pv) Combo (Access Bio, Inc., Somerset, NJ, USA) RDT. However, microscopical blood film examination confirmed infection with *P. malariae*. The initial parasite density count was 5380 P/µL. This initial count was performed by an experienced and WHO-certified microscopist. The patient received treatment in accordance with the national guideline [14]. Three milliliters of blood was collected and transported along with the slides to the Emerging Infections & Parasitology Laboratory (EIPL) of the icddr,b in Dhaka. At the EIPL, a parasitemia of 5930P/µL was re-counted in a thin blood film by a second microscopist who is also specialized in this field. The sample was tested for LDH and aldolase antigenic target using three brands of RDTs (1) STANDARD Q Malaria P.f/Pan Ag (SD biosensor, Cheongju-si, Republic of Korea), (2) Parascreen^TM^ Rapid Malaria Pf/Pan (Zephyr Biomedical Systems, Goa, India), and (3) BinaxNOW^®^ Malaria Test (Inverness Medical Innovations, Inc., Waltham, MA, USA). The sample tested negative for PfHRP2 (Figure 1a–c) and negative for both pLDH and aldolase (Figure 1a,d).

Initially, to rule out the prozone effect, the sample was diluted with uninfected fresh erythrocytes, and the RDT results remained negative (Figure 1e–g). An archived sample collected from the same geographical location (AUHC) with parasitemia of 5040 P/µL was used as a positive control in the RDT examination and gave a positive result (Figure 1h). A mono-infection with *P. malariae* was confirmed using 18S ribosomal RNA (rRNA) *Plasmodium* sp. and species-specific PCRs [15]. The PCR result was validated by a plasmid DNA control of *P. malariae* (Catalog No. MRA-179). On the other hand, a negative result for CareStart™ Malaria HRP2/pLDH (Pf/Pv) Combo (Access Bio, Inc., Somerset, NJ, USA) defined no cross reactivity of the sample with *P. falciparum* or *P. vivax* antigenic target (Figure 1c). 

To prove the absence of genetic variability and address the discordance between RDT and microscopy/PCR, the protein-coding region of LDH gene of *P. malariae* of this sample was sequenced. Talman et al. [16] showed that genetic variability has been found in the LDH gene of non-falciparum human malaria species. On the other hand, genetic variation in the aldolase gene has been ruled out as a possible reason for variation of RDT sensitivity [17]. The LDH gene sequence of the sample (GenBank Accession No: MN998417) matched the reference gene (XM029006607). Quantification by ELISA of the LDH enzyme released in the sample could have been helpful but was a limitation in this case due to the lack of sufficient material.

## 3. Discussion

Very recently, a negative RDT result was reported in a *P. malariae* case imported from West Africa to China [18]. The suboptimal performance of the RDTs for detecting the neglected *Plasmodium* species including *P. malariae* was also documented [19]. Aldolase RDTs do not react reliably with *P. malariae* parasites because of low concentrations of this enzyme [20], whereas the sensitivity of pLDH-based RDTs ranges between 21.4 and 45.2% for the diagnosis of *P. malariae* [12]. 

One explanation for the poor sensitivity of the RDTs is the lower affinity of some monoclonal antibodies for the parasite [21]. In comparison with *P. falciparum* or *P. vivax*, parasitemia is lower in *P. malariae,* usually occurring as mixed rather than mono infections and therefore can be undetectable by RDTs and even by microscopy [22,23]. However, symptomatic *P. malariae* cases also went undetected by RDTs, and higher parasitemia definitely could not assure the accuracy of the RDTs [24,25,26]. The reason for the poor sensitivity of RDTs in the detection of this parasite is yet to be determined [27], and in-depth research on the antigenic nature of this parasite is urgently needed. The clinical presentation of *P. malariae* mono-infection is not rare in this region [2]. However, depending on RDTs and complexity in blood smears, cases may be misdiagnosed. Recently, a few studies have been focusing on the development of diagnostic markers, i.e., the sensitive monoclonal antibody selection of LDH of *P. malariae* to increase the sensitivity of the RDTs and the testing of recombinant proteins of *P. malariae* merozoite surface protein 1 as a promising diagnostic marker as well as a vaccine target [28,29]. Nevertheless, the small number of diagnosed *P. malariae* cases hinders the evaluation and development of RDTs for this species. 

Woodford et al. [30] conducted an induced blood-stage malaria (IBSM) model experiment to study *P. malariae* infection in humans. Although in this study, the pLDH level was correlated with increased parasitemia and had the appropriate attributes for a screening test, the pLDH RDT result was negative. Thus, this rapid diagnostic kit appears to be too insensitive for this species, and case detection, true estimates of disease burden, and elimination efforts remain a challenge. This emphasizes again that even though there is a quantifiable pLDH antigen, RDTs can readily fail to detect this parasite.

C-reactive protein (CRP) acts as a prognosis marker of disease severity in *P. falciparum* and *P. vivax* malaria. The CRP level increases with the parasitemia, representing a nonspecific host response, and maintains consistent correlation with disease progression [31]. In this IBSM model, however, *P. malariae* showed different dynamics. It presented a relatively delayed lag period in pLDH positivity with respect to parasite appearance after inoculation. This provides a clear difference in interspecies pLDH biomarker dynamics. The pLDH-based RDT is designed to target the same pLDH level regardless of the malaria species. These findings can shed light on the development of species-specific RDT kits. 

Studies have shown that PfHRP2 has a relatively long half-life (2–4 weeks), favoring the development of anti-PfHRP2 antibodies in the host blood and leading to false negative results The current authors also proposed that the similar blocking effect may hinder the performance of LDH or aldolase [32]. A 72 hour developmental cycle helps the blood stage of *P. malariae* to persist for a very long time in host facilities. Thus, circulating LDH antigen released by parasites assists in the formation of host antibodies [33]. This anti-LDH immune complex may possibly inhibit the detection of pLDH by RDT. Malaria elimination approaches need to focus on understanding the biology and transmission dynamics of less-appreciated parasite species like *P. malariae* [34].

## 4. Conclusions

In endemic areas, malaria case management mainly focuses on the most relevant *Plasmodium* species, namely, *P. falciparum* and *P. vivax.* Neglected *Plasmodium* species like *P. malariae* are often overlooked. In our case, the RDT diagnosis of a symptomatic patient with relatively high parasitemia failed. It is evident that no reliable screening kits/RDTs are available for the diagnosis of *P. malariae*. Diagnosis with microscopy and PCR is often not possible in endemic rural settings. Since (mono-) infections of *P. malariae* are not uncommon, proper diagnosis may reveal more subclinical malaria cases in endemic countries. Lately, the paradigm shift from malaria control to malaria elimination in Bangladesh underscores the importance of having an efficient method of point-of-care testing for *P. malariae*. We recommend that the health care team in endemic areas be aware of RDT failings and propose that additional microscopical or molecular diagnostic tools might be needed to avoid false negative results. 

## Data Availability

Not applicable.
